# Global burden and projections of stroke and its subtypes attributable to high alcohol use during 1990–2021: insights from the global burden of disease study 2021

**DOI:** 10.3389/fneur.2025.1653790

**Published:** 2025-09-08

**Authors:** Fangfang Zhan, Yifan Chen, Hong Qiu, Yimin Chen, Shuping Lin, Liqiong Zhan, Jun Ni

**Affiliations:** ^1^Department of Rehabilitation, The First Affiliated Hospital of Fujian Medical University, Fuzhou, Fujian, China; ^2^Department of Rehabilitation, National Regional Medical Center, Binhai Campus of the First Affiliated Hospital, Fujian Medical University, Fuzhou, Fujian, China; ^3^Fujian Orthopedic Bone and Joint Disease and Sports Rehabilitation Clinical Medical Research Center, Fuzhou, Fujian, China; ^4^College of Integrated Traditional Chinese, Fujian University of Traditional Chinese Medicine, Fuzhou, Fujian, China; ^5^Fuzhou Traditional Chinese Medicine Hospital, Fuzhou, Fujian, China; ^6^Department of Rehabilitation, Affiliated Rehabilitation Hospital of Fujian University of Traditional Chinese Medicine, Fuzhou, Fujian, China; ^7^Xianyou County General Hospital, Putian, Fujian, China

**Keywords:** GBD, stroke, high alcohol use, disability-adjusted life years, sociodemographic index, estimated annual percentage change

## Abstract

**Background:**

Stroke represents a critical public health challenge with far-reaching global implications for human health. In this study, we seek to characterize the global trends in the burden of stroke attributable to high alcohol use from 1990 to 2021 and investigate its associations with socioeconomic status.

**Methods:**

Data on the burden of stroke attributable to high alcohol use were derived from the Global Burden of Disease (GBD) study. The main evaluation indicators include mortality, disability-adjusted life years (DALYs), age-standardized mortality rates (ASMR), and age-standardized DALY rates (ASDR). Trends in disease burden across genders, age groups, and regions were analyzed. Decomposition, frontier, and forecasting analyses were also conducted for the disease.

**Results:**

In 2021, stroke attributable to high alcohol consumption remain a substantial global health burden, accounting for 253,625 deaths (95% UI: 53,445–506,300) and 6,003,243 DALYs (95% UI: 1,323,435–11,423,164) worldwide, respectively. ASMR and ASDR for stroke attributable to high alcohol consumption show a global decline, with an EAPC of −1.81 (95% CI: −1.88 to −1.75) for ASMR and −1.63 (95% CI: −1.70 to −1.56) for ASDR. Findings reveal that in global, high, high-middle and middle SDI regions, epidemiological changes are the primary drivers of decline in mortality and DALYs for disease burden. By 2030, the ASMR for high alcohol use-attributable stroke is projected to decline from 4.3 per 100,000 in 2021 to 4.15 per 100,000, while the ASDR is forecast to decrease from 98 per 100,000 in 2021 to 95 per 100,000.

**Conclusion:**

In summary, ASMR and ASDR for stroke attributable to high alcohol consumption decline globally, and in most regions from 1990 to 2021. Relevant countries and institutions should continue to attach great importance to the impact of this disease and formulate targeted policies.

## 1 Introduction

Stroke stands as a critical public health challenge with far-reaching global implications for human health. As the third leading cause of death and disability worldwide, it places a substantial burden on individuals, families, and healthcare systems (1). From 1990 to 2019, stroke’s impact on disability-adjusted life years (DALYs) grew increasingly pronounced, accounting for 6.55 million deaths and 143 million DALYs globally (2). This trend reflects a 41.4% rise in DALYs over the period, driven by aging populations, escalating chronic risk factors, and a shift in burden from acute mortality to long-term disability. Medical care and post-stroke rehabilitation impose a heavy burden on healthcare systems and society as a whole (3). This burden is exacerbated by long-term care needs: 30–40% of survivors require ongoing assistance with daily activities, which further strains family resources and healthcare budgets. To effectively alleviate these burdens, intensified focus on stroke epidemiological surveillance, prevention, and treatment research is essential.

Alcohol consumption constitutes a pervasive behavioral risk factor affecting both individual and public health. It elevates the risk of unintentional and intentional injuries, as well as non-communicable diseases (NCDs) such as cancer, gastrointestinal disorders, and cardiovascular diseases (CVD), along with infectious diseases including tuberculosis and pneumonia (4). While previous cohort studies and meta-analyses have explored the potential association between alcohol intake and incident CVD, the characterization of their dose–response relationship remains inconsistent across investigations (5, 6). Piano et al. highlighted that heavy alcohol consumption—defined as binge drinking or an average of ≥3 drinks per day—has been consistently linked to adverse outcomes across all examined CVD subtypes (7). Alcohol consumption is a prominent modifiable lifestyle risk factor for stroke, contributing to both ischemic and hemorrhagic subtypes through well-characterized biological mechanisms (8). From 1990 to 2019, the global burden of stroke-related DALYs attributable to high alcohol consumption increased significantly, despite a concurrent decline in age-standardized DALY rates (2). According to the Global Burden of Disease Study 2019, total stroke DALYs rose from 91.5 million to 125 million, with high alcohol consumption emerging as a key risk factor driving this growth in absolute burden. However, data on deaths resulting from high alcohol use-attributable stroke, as well as the geographical distribution of such deaths across global regions, remain undissected. With societal and economic development, advances in medical standards, and the implementation of alcohol restriction policies worldwide, we anticipate a gradual decline in stroke deaths attributable to high alcohol consumption. In this study, we aimed to characterize global trends in the burden of high alcohol consumption-attributable stroke (with a specific focus on mortality) from 1990 to 2021, examine their associations with socioeconomic status, and investigate projected trends over the next decade.

## 2 Materials and methods

### 2.1 Data resource

In this study, data on the burden of stroke and its subtypes attributable to high alcohol use were derived from the Global Burden of Disease (GBD) study^1^. Developed by GBD collaborators, this initiative provides a systematic assessment of mortality, prevalence, years lived with disability, and comparative risks across countries and territories. We leveraged publicly accessible data from the GBD 2021 database to analyze trends in mortality and DALYs related to stroke and its subtypes (including ischemic stroke and intracerebral hemorrhage), specifically those attributable to high alcohol consumption, over the period from 1990 to 2021. Stroke is defined in accordance with WHO clinical criteria as a disorder marked by the rapid onset of clinical manifestations of (typically focal) cerebral functional impairment, persisting for more than 24 h or resulting in death (9). Ischemic stroke is defined as an episode of neurological impairment caused by focal infarction in the cerebrum, spinal cord, or retina. Intracerebral hemorrhage was characterized as a form of stroke involving a localized accumulation of blood within the brain that is not trauma-induced. Alcohol exposure is defined as the grams of pure alcohol consumed daily by individuals who had consumed at least one alcoholic beverage in previous years. To quantify the proportion of disease-specific burden attributable to each risk factor, the GBD study integrated a comparative risk assessment approach (10). Mortality rates were estimated using data from vital registration sources, as defined by the International Classification of Diseases (ICD) codes, including ICD-10, ICD-9, and ICD-8 (11). Ethical approval and informed consent were waived for this study, as the GBD data utilized are publicly accessible and contain no identifiable participant information, thus eliminating the need for individual consent or institutional review board approval.

### 2.2 Measures of burden

Measures of disease burden included mortality and DALYs from 1990 to 2021. Mortality rates were primarily estimated using the cause-of-death ensemble model. DALYs, defined as the total healthy years lost from disease onset to death, serve as a critical parameter for assessing disease burden, as they integrate both mortality and morbidity components into a single metric.

### 2.3 Sociodemographic index

The sociodemographic index (SDI), a composite indicator reflecting the developmental level of a country or region, is constructed based on lag-distributed per capita income and average educational attainment. Strong correlations are observed between SDI and health outcomes, including mortality and DALYs. SDI ranges from 0 (representing the lowest development) to 1 (representing the highest development). To explore the association between stroke burden and SDI, we classified 204 countries and 21 regions into five groups—low, low-middle, middle, high-middle, and high—based on their SDI values.

### 2.4 Decomposition analysis

In this study, we conducted a decomposition analysis to quantify the contributions of aging, population growth, and epidemiological changes to stroke-related mortality and DALYs across various countries and regions from 1990 to 2021.

### 2.5 Frontier analysis

We employed frontier analysis to quantify the relationship between high alcohol use-attributable stroke and SDI. This approach generates a non-linear frontier that represents the minimum achievable burden of high alcohol use-attributable stroke at a given level of development. The “effective difference” is defined as the gap between a country’s observed disease burden and its theoretically achievable burden based on its SDI.

### 2.6 Forecasting stroke burden

The Auto-Regressive Integrated Moving Average (ARIMA) model—an amalgamation of an autoregressive (AR) component and a moving average (MA) model—is a widely utilized technique in epidemiology for forecasting time-series data (12). Formally denoted as ARIMA (p, d, q), the model defines p as the autoregressive order, d as the degree of differencing required to achieve stationarity, and q as the moving average order. In this study, we constructed an ARIMA model to forecast the burden of high alcohol use-attributable stroke from 2020 to 2030.

### 2.7 Statistical analyses

Mortality and DALYs from 1990 to 2021 were quantified as both absolute numbers and age-standardized rates (per 100,000 population). Stroke-related mortality and DALYs attributable to high alcohol use were stratified by sex, age, and year. Multidimensional analyses encompassed global and regional distribution patterns, gender disparities, temporal trends via estimated annual percentage change (EAPC), disease-specific burden, age-specific distributions, and correlations with the SDI. EAPCs were determined using the regression model: *Y* = *α* + βX + *ε*, where Y represents the natural logarithm of the age-standardized rate, X is the calendar year, and ε is the error term (13). Statistical significance was defined as *p*-values < 0.05. All statistical analyses and visualizations were performed using R (version 4.3.2).

## 3 Results

### 3.1 Global burden of stroke attributable to high alcohol use

In 2021, the stroke attributable to high alcohol use remained a substantial global health burden, accounting for 253,625 (95% UI: 53445–506,300) deaths and 6,003,243 (95% UI: 1323435–11,423,164) DALYs worldwide. The age-standardized rates of mortality (ASMR) decreased from 7.2/per 100,000 (95% UI: 1.4–14.66) in 1990 to 4.3/per 100,000 (95% UI: 1–8.39) in 2021, with an EAPC of −1.81 (95% CI: −1.88 to −1.75) (Table 1). Concurrently, the age-standardized rates of DALYs (ASDR) reduced from 154.83/per 100,000 (95% UI: 33.98–299.48) in 1990 to 97.89/per 100,000 (95% UI: 23.83–187.71) in 2021, with an EAPC of −1.63 (95% CI: −1.7 to −1.56) (Table 2). These trends collectively reflect a consistent global reduction in mortality from 1990 to 2021.

**Table 1:** Mortality cases, age-standardized rates of mortality (ASMR, per 100,000) of stroke attributable to high alcohol use from 1990 to 2021, and estimated annual percentage changes (EAPCs) in age-standardized rates over the same period.

Region and gender	1990		2021		1990–2021
	Death (*10^2^; 95% UI)	ASMR (95% UI)	Death (*10^2^; 95% UI)	ASMR (95% UI)	EAPC of ASMR (95% CI)
Overall	2536.25 (534.45–5,063)	7.2 (1.4–14.66)	3607.2 (859.63–7018.13)	4.3 (1–8.39)	−1.81 (−1.88 to −1.75)
Sex					
Male	1897.67 (424.92–3617.52)	12.38 (2.55–24.7)	3032.39 (767.08–5818.12)	8.14 (1.91–15.74)	−1.47 (−1.53 to −1.41)
Female	638.57 (89.03–1367.82)	3.35 (0.46–7.25)	574.8 (93.88–1214.23)	1.22 (0.2–2.59)	−3.53 (−3.66 to −3.39)
SDI					
High SDI	724.56 (101.48–1518.35)	6.5 (0.91–13.6)	598.61 (102.68–1230.14)	2.49 (0.45–5.05)	−3.28 (−3.4 to −3.17)
High-middle SDI	1039.54 (202.52–2089.98)	11.56 (2.06–23.67)	1181.28 (232.65–2403.63)	6.03 (1.19–12.28)	−2.45 (−2.69 to −2.22)
Middle SDI	557.7 (126.06–1087.03)	5.97 (1.39–11.48)	1319.77 (335.28–2462.91)	5.24 (1.28–9.89)	−0.33 (−0.44 to −0.22)
Low-middle SDI	132.4 (29.4–260.33)	2.36 (0.57–4.74)	351.87 (84.73–686.05)	2.56 (0.63–5.02)	0.37 (0.28 to 0.46)
Low SDI	77.43 (14.48–153.07)	3.84 (0.79–7.42)	151.66 (35.17–295.2)	3.38 (0.82–6.46)	−0.51 (−0.76 to −0.26)

**Table 2:** DALYs cases, age-standardized rates of DALYs (ASDR, per 100,000) of stroke attributable to high alcohol use from 1990 to 2021, and estimated annual percentage changes (EAPCs) in age-standardized rates over the same period.

Region and gender	1990		2021		1990–2021
	DALY (*10^2^; 95% UI)	ASDR (95% UI)	DALY (*10^2^; 95% UI)	ASDR (95% UI)	EAPC of ASDR (95% CI)
Overall	60032.43 (13234.35–114231.64)	154.83 (33.98–299.48)	84372.72 (20564.66–161013.15)	97.89 (23.83–187.71)	−1.63 (−1.7 to −1.56)
Sex					
Male	47284.17 (11383.51–88140.72)	265.85 (59.02–505.77)	72857.69 (18362.2–137238.06)	181.55 (44.5–345.6)	−1.34 (−1.4 to −1.28)
Female	12748.25 (2157.21–26548.62)	62.51 (10.14–131.75)	11515.03 (2161.6–24083.19)	24.89 (4.72–51.84)	−3.3 (−3.44 to −3.16)
SDI					
High SDI	14644.96 (2467.54–29431.72)	132.55 (22.99–266.22)	12077.72 (2203.16–24721.01)	57.25 (10.71–115.15)	−2.88 (−2.99 to −2.77)
High-middle SDI	24560.18 (5289.64–47123.04)	250.7 (51.62–489.75)	26413.1 (5874.84–52467.39)	134.48 (30.1–266.02)	−2.39 (−2.63 to −2.14)
Middle SDI	14969.78 (3021–29023.52)	141.18 (31.07–275.13)	32090.61 (8240.9–59606.06)	118.52 (30.96–219.97)	−0.45 (−0.55 to −0.34)
Low-middle SDI	3665.7 (760.34–7250.76)	58.03 (12.79–114.64)	9604.69 (2208.5–18499.54)	64.04 (14.92–123.09)	0.43 (0.34 to 0.52)
Low SDI	2090.13 (353.04–4302.54)	91.01 (17.08–181.35)	4100.29 (872.07–8171.97)	79.17 (18.21–153.35)	−0.58 (−0.83 to −0.33)

### 3.2 Global trends by gender and age

**Figure 1:**
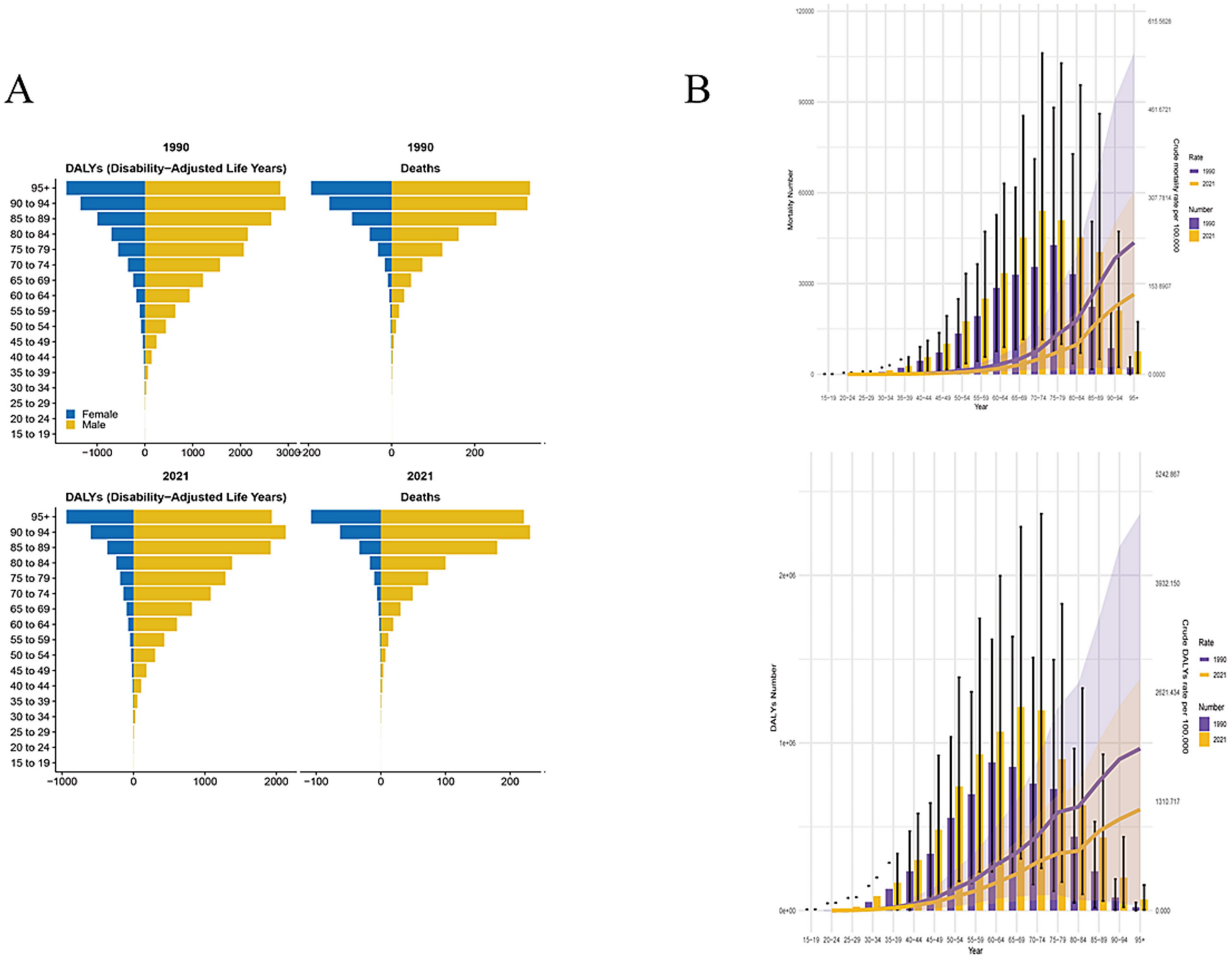
Trends in stroke attributable to higher alcohol use mortality during 1990–2021. **(A)** The burden of disease across different age groups. **(B)** The change in the number and rate of deaths and DALYs across different age groups from 1990 to 2021.

When stratified by gender, mortality for stroke attributable to high alcohol use declined in male and female by 2021 (Tables 1, 2). These trends were mirrored in both ASMR and ASDR. The decline observed in females is more pronounced than that in males, accompanied by a higher EAPC. Figure 1A illustrates the burden of stroke attributable to high alcohol use across different age groups in 1990 and 2021. In general, the number of deaths and DALYs were higher in males aged 30 and older than in females. Additionally, we observed that the death cases increase with age, highlighting the need for greater attention to disease incidence among the elderly population. Compared with 1990, both the death cases and DALYs increased substantially by 2021. Then, we found that the highest global burden of deaths of stroke attributable to high alcohol use occurred in individuals aged 70–74 years, whereas the DALYs were observed in the 65–74 age group (Figure 1B). The majority of cases occur in individuals aged 40 to 95 years. However, both ASMR and ASDR showed declining trends, indicating advancements in healthcare and the effective implementation of policies.

### 3.3 Global trends by region and socioeconomic status

**Figure 2:**
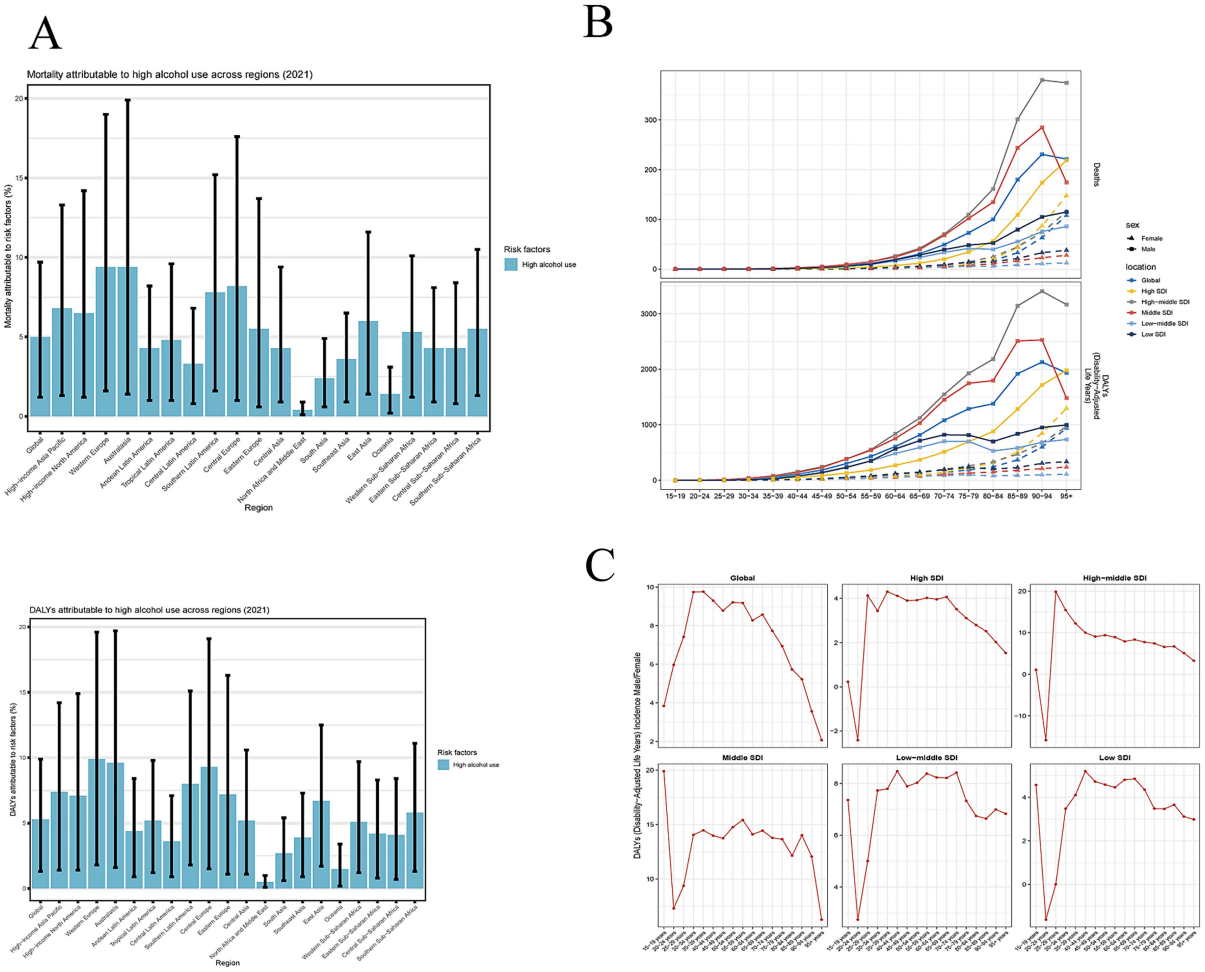
Trends in stroke attributable to higher alcohol use mortality across different regions. **(A)** The percentage of high alcohol use in the total risk factors by regions. **(B)** The change in mortality and DALYs for males and females across different age groups in different SDI regions. **(C)** The age-specific male-to-female rate ratios across different age groups.

At regional and national levels, countries/territories in the High-income Asia Pacific, Western Europe, and Southern Latin America regions exhibit the most pronounced declines in ASMR and ASDR trends, as measured by EAPC (Supplementary Tables 1, 2). The regions with the least significant decline include High-income North America, Southern Sub-Saharan Africa, and Central Sub-Saharan Africa. Additionally, our findings indicate that high alcohol consumption accounts for nearly 10% of stroke-related deaths among all risk factors in Western Europe and Australasia--the two regions with the highest proportion of such deaths attributable to high alcohol consumption (Figure 2A). In contrast, the contribution of high alcohol consumption to deaths is the lowest in North Africa, the Middle East, and Oceania.

Countries in high and high-middle SDI groups show significant declines in ASMR and ASDR, as quantified by EAPC (Tables 1, 2). Middle and low SDI groups exhibit slightly decreased. However, the low-middle SDI group show an increasing trend, with a positive EAPC. We hypothesize that population growth and inadequate healthcare conditions may be the primary factors contributing to this phenomenon. When stratified by gender and age, males in the high-middle SDI group exhibit the highest death rates and DALYs rates, particularly among those aged 65 years and older, followed by males in the middle SDI (Figure 2B). The mortality rates of both groups also exceed the global average. In the female group, those in the high SDI category have the highest death rates and DALY rates. Additionally, a significant increase in the death cases is observed among elderly individuals aged 85 and above. The death cases among females in the middle, low-middle, and low SDI groups shows a relatively flat trend. Figure 2C present the age-specific male-to-female rate ratios, illustrating sex differences in mortality and DALYs across different age groups. Globally, males aged 35–39 have a > 9.5-fold higher DALY rate than females in the same age group; in the high SDI group, males aged 25–29 show a > 4-fold higher rate than females; in the high-middle SDI group, males aged 25–29 have a 20-fold higher rate; in the middle SDI group, males aged 15–19 exhibit a 20-fold higher rate; in the low-middle SDI group, males aged 40–44 have a > 8-fold higher rate; and in the low SDI group, males aged 40–44 show a > 5-fold higher rate than females. The above findings indicate that the incidence rate in men is consistently higher than that in women.

### 3.4 The burden and temporal trends of stroke subtypes attributable to high alcohol use

In 2021, the ischemic stroke attributable to high alcohol use cause 181,949 (95% UI: 24601–455,500) mortality and 3,870,519 (95% UI: 603265–9,620,467) DALYs worldwide. The ASMR decrease from 4.15/per 100,000 (95% UI: 0.43–10.2) in 1990 to 2.21/per 100,000 (95% UI: 0.29–5.53) in 2021, with an EAPC of −2.23 (95% CI: −2.31 to −2.15) (Table 3). Concurrently, the ASDR reduce from 76.45/per 100,000 (95% UI: 9.55–186.84) in 1990 to 45.44/per 100,000 (95% UI: 6.99–112.92) in 2021, with an EAPC of −1.9 (95% CI: −1.98 to −1.81) (Table 4). For intracerebral hemorrhage, the burden comprises 118,617 (95% UI: 1828–251,563) deaths and 4,566,754 (95% UI: 135897–9,388,359) DALYs worldwide. The ASMR for this subtype decrease from 3.05/100,000 (95% UI: 0.05–6.51) in 1990 to 2.09/per 100,000 (95% UI: 0.06–4.28) in 2021, with an EAPC of −1.32 (95% CI: −1.4 to −1.24) (Table 3). Concurrently, the ASDR reduce from 78.37/per 100,000 (95% UI: 1.25–164.48) in 1990 to 52.45/per 100,000 (95% UI: 1.54–108) in 2021, with an EAPC of −1.38 (95% CI: −1.46 to −1.31) (Table 4). These results reflect a consistent global downward trend in both mortality and disability burden of stroke subtypes over the study period.

**Table 3:** Mortality cases, age-standardized mortality rates (ASMR, per 100,000) for stroke subtypes attributable to high alcohol use from 1990 to 2021, and EAPCs in these age-standardized rates over the same period.

Region and gender	1990		2021		1990–2021
	Death (*10^2^; 95% UI)	ASMR (95% UI)	Death (*10^2^; 95% UI)	ASMR (95% UI)	EAPC of ASMR (95% CI)
Ischemic stroke					
Overall	1350.08 (148.27–3310.38)	4.15 (0.43–10.2)	1819.49 (246.01–4,555)	2.21 (0.29–5.53)	−2.23 (−2.31 to −2.15)
Sex					
Male	923.03 (115.3–2255.96)	6.83 (0.76–16.79)	1461.64 (204.16–3641.27)	4.13 (0.54–10.31)	−1.78 (−1.85 to −1.71)
Female	427.05 (34.95–1074.38)	2.32 (0.18–5.85)	357.85 (35.49–919.72)	0.76 (0.08–1.94)	−3.92 (−4.07 to −3.77)
SDI					
High SDI	511.58 (51.59–1219.37)	4.57 (0.46–10.91)	398.92 (49.2–961.3)	1.56 (0.2–3.76)	−3.68 (−3.82 to −3.54)
High-middle SDI	590.1 (59.01–1462.83)	6.95 (0.66–17.43)	686.45 (81.46–1732.75)	3.51 (0.41–8.86)	−2.58 (−2.79 to −2.36)
Middle SDI	178.59 (29.88–448.96)	2.14 (0.34–5.4)	542.96 (83.86–1376.58)	2.26 (0.33–5.72)	0.27 (0.15 to 0.39)
Low-middle SDI	44.92 (6.72–122.69)	0.93 (0.13–2.55)	134.63 (18.87–358.44)	1.08 (0.14–2.87)	0.59 (0.5 to 0.68)
Low SDI	21.91 (3.48–60.33)	1.32 (0.21–3.59)	53.97 (8.92–143.37)	1.42 (0.22–3.72)	0.19 (0.03 to 0.35)
Intracerebral hemorrhage					
Overall	1186.17 (18.28–2515.63)	3.05 (0.05–6.51)	1787.71 (49.42–3664.4)	2.09 (0.06–4.28)	−1.32 (−1.4 to −1.24)
Sex					
Male	974.65 (16.29–2050.89)	5.55 (0.09–11.77)	1570.76 (39.36–3210.61)	4.01 (0.1–8.19)	−1.12 (−1.21 to −1.04)
Female	211.52 (4.03–462.93)	1.02 (0.02–2.25)	216.95 (10.06–469.89)	0.47 (0.02–1.01)	−2.77 (−2.89 to −2.65)
SDI					
High SDI	212.98 (5.27–448.75)	1.93 (0.05–4.06)	199.69 (5.55–425.05)	0.92 (0.03–1.95)	−2.49 (−2.56 to −2.43)
High-middle SDI	449.43 (7.29–951.96)	4.61 (0.08–9.8)	494.83 (10.39–1051.29)	2.52 (0.05–5.36)	−2.27 (−2.54 to −2.01)
Middle SDI	379.11 (4.84–814.83)	3.83 (0.05–8.27)	776.81 (18.32–1579.14)	2.97 (0.07–6.08)	−0.71 (−0.83 to −0.59)
Low-middle SDI	87.49 (3.97–193.55)	1.43 (0.07–3.14)	217.23 (7.14–467.24)	1.48 (0.05–3.19)	0.22 (0.11 to 0.33)
Low SDI	55.52 (1.59–127)	2.52 (0.08–5.66)	97.69 (3.16–217.97)	1.97 (0.07–4.36)	−0.94 (−1.24 to −0.64)

**Table 4:** DALYs cases, age-standardized rates of DALYs (ASDR, per 100,000) of stroke subtypes attributable to high alcohol use from 1990 to 2021, and estimated annual percentage changes (EAPCs) in age-standardized rates over the same period.

Region and gender	1990		2021		1990–2021
	DALY (*10^2^; 95% UI)	ASDR (95% UI)	DALY (*10^2^; 95% UI)	ASDR per (95% UI)	EAPC of ASDR (95% CI)
Ischemic stroke					
Overall	27770.06 (3705.7–68000.95)	76.45 (9.55–186.84)	38705.19 (6032.65–96204.67)	45.44 (6.99–112.92)	−1.9 (−1.98 to −1.81)
Sex					
Male	20224.33 (3004.36–48990.08)	126.54 (17.09–306.98)	32162.99 (5238.03–79017.47)	83.24 (13.09–205.04)	−1.52 (−1.6 to −1.45)
Female	7545.73 (754.58–19176.04)	38.15 (3.78–96.69)	6542.19 (798.35–16643.09)	14.01 (1.72–35.67)	−3.59 (−3.74 to −3.44)
SDI					
High SDI	9269.41 (1048.99–22042.86)	82.25 (9.4–195.85)	7692.36 (1135.61–18367.78)	34.21 (5.38–82.08)	−3.04 (−3.17 to −2.9)
High-middle SDI	12474.36 (1476.36–30562.45)	132.37 (14.89–326.16)	14309.23 (2067.94–35799.62)	71.97 (10.31–179.89)	−2.38 (−2.61 to −2.14)
Middle SDI	4358.38 (783.67–10843.85)	45.19 (7.81–112.57)	12179.16 (2104.17–30,453)	46.74 (7.8–117.08)	0.22 (0.09 to 0.35)
Low-middle SDI	1089.14 (168.7–3000.43)	19.48 (2.97–53.3)	3193.07 (495.8–8508.61)	23.12 (3.46–61.77)	0.63 (0.56 to 0.7)
Low SDI	520.95 (88.58–1424.2)	26.48 (4.27–71.78)	1282.59 (221.58–3379.06)	28.5 (4.88–75.17)	0.17 (0.02 to 0.33)
Intracerebral hemorrhage					
Overall	32262.37 (504.7–67553.4)	78.37 (1.25–164.48)	45667.54 (1358.97–93883.59)	52.45 (1.54–108)	−1.38 (−1.46 to −1.31)
Sex					
Male	27059.85 (453.65–56371.01)	139.31 (2.35–291.31)	40694.7 (1146.96–83120.57)	98.31 (2.7–201.48)	−1.18 (−1.26 to −1.1)
Female	5202.52 (90.36–11223.13)	24.36 (0.45–52.93)	4972.84 (205.78–10696.95)	10.88 (0.44–23.38)	−2.89 (−3.02 to −2.77)
SDI					
High SDI	5375.56 (123.49–10982.5)	50.3 (1.15–102.3)	4385.36 (127.84–9121.87)	23.04 (0.67–47.31)	−2.64 (−2.71 to −2.57)
High-middle SDI	12085.81 (193.11–25046.03)	118.33 (1.96–246.36)	12103.87 (264.6–25319.51)	62.52 (1.34–130.64)	−2.4 (−2.66 to −2.13)
Middle SDI	10611.4 (143.33–22608.07)	96 (1.28–204.89)	19911.45 (544.05–40218.9)	71.78 (1.89–145.01)	−0.81 (−0.92 to −0.7)
Low-middle SDI	2576.56 (103.82–5800.85)	38.55 (1.68–85.56)	6411.62 (199.38–13843.13)	40.92 (1.3–88.29)	0.33 (0.22 to 0.44)
Low SDI	1569.18 (34.53–3665.9)	64.54 (1.76–149.6)	2817.7 (74.79–6256.91)	50.67 (1.59–112.81)	−0.94 (−1.24 to −0.65)

**Figure 3:**
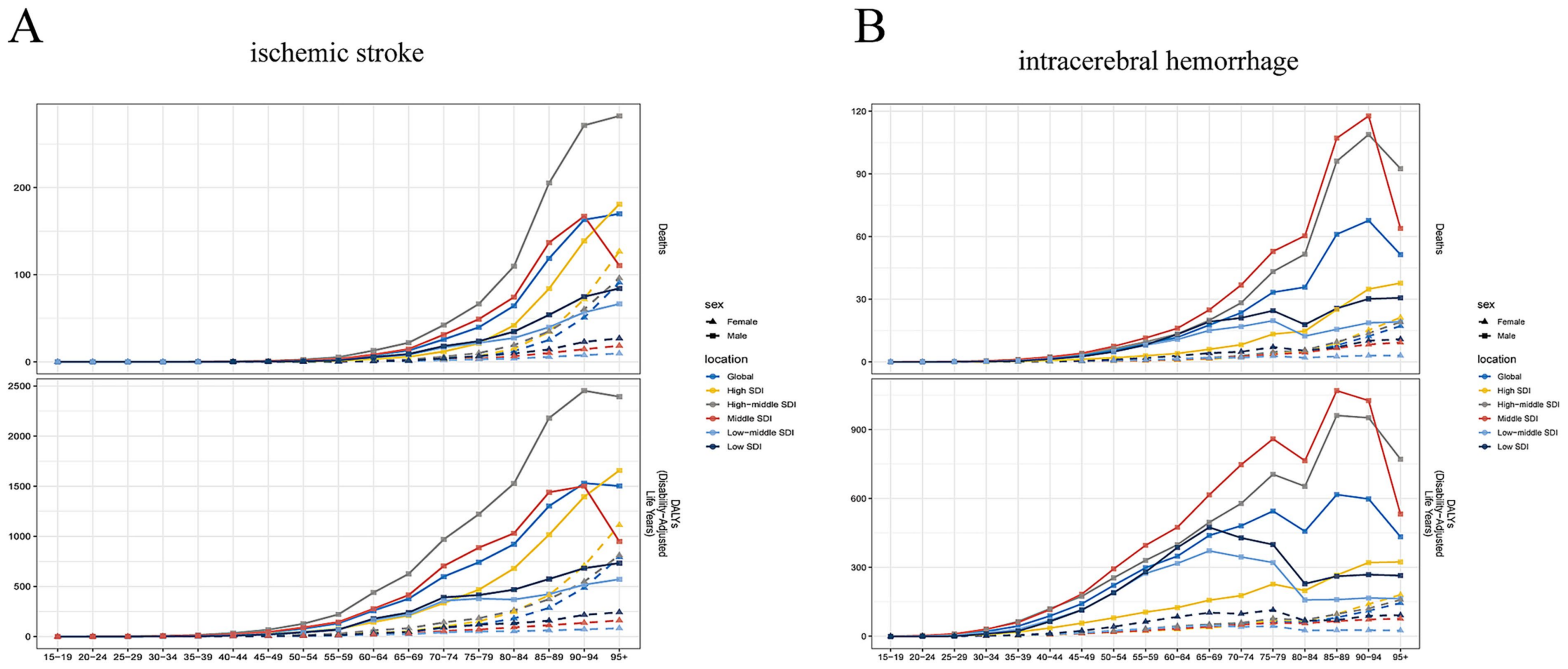
The burden of stroke subtypes attributable to high alcohol use. **(A)** The change in mortality and DALYs of ischemic stroke for males and females across different age groups in different SDI regions. **(B)** The change in mortality and DALYs of intracerebral hemorrhage for males and females across different age groups in different SDI regions.

For ischemic stroke, the top three regions with the most substantial declines in ASMR and ASDR are High-income Asia Pacific, Western Europe, and Southern Latin America (Supplementary Tables 3, 4). However, an upward trend is observed in some regions, including Southeast Asia and South Asia. When stratified by gender and age, males with ischemic stroke in the high-middle SDI group have the highest death rates and DALYs rates, particularly among those aged 65 years and older. This trend is followed by males in the middle SDI group (Figure 3A). In the female group, those in the high SDI category have the highest death rates and DALY rates, particularly among those aged 85 years and older. The death cases among females in the middle, low-middle, and low SDI groups exhibits a relatively stable trend. For intracerebral hemorrhage, the regions demonstrating the most pronounced declines in ASMR and ASDR are Southern Latin America, North Africa and the Middle East, and High-income Asia Pacific (Supplementary Tables 3, 4). However, an upward trend is also observed in some regions, including Southeast Asia, South Asia, and High-income North America. When stratified by gender and age, males with intracerebral hemorrhage in the middle SDI group have the highest death rates and DALYs rates, particularly among those aged 55 years and older. This trend is followed by males in the High-middle SDI group (Figure 3B). In the female group, individuals in the high SDI category exhibit the highest death rates and DALY rates, with this trend being particularly pronounced among those aged 85 years and older.

### 3.5 Decomposition analysis

**Figure 4:**
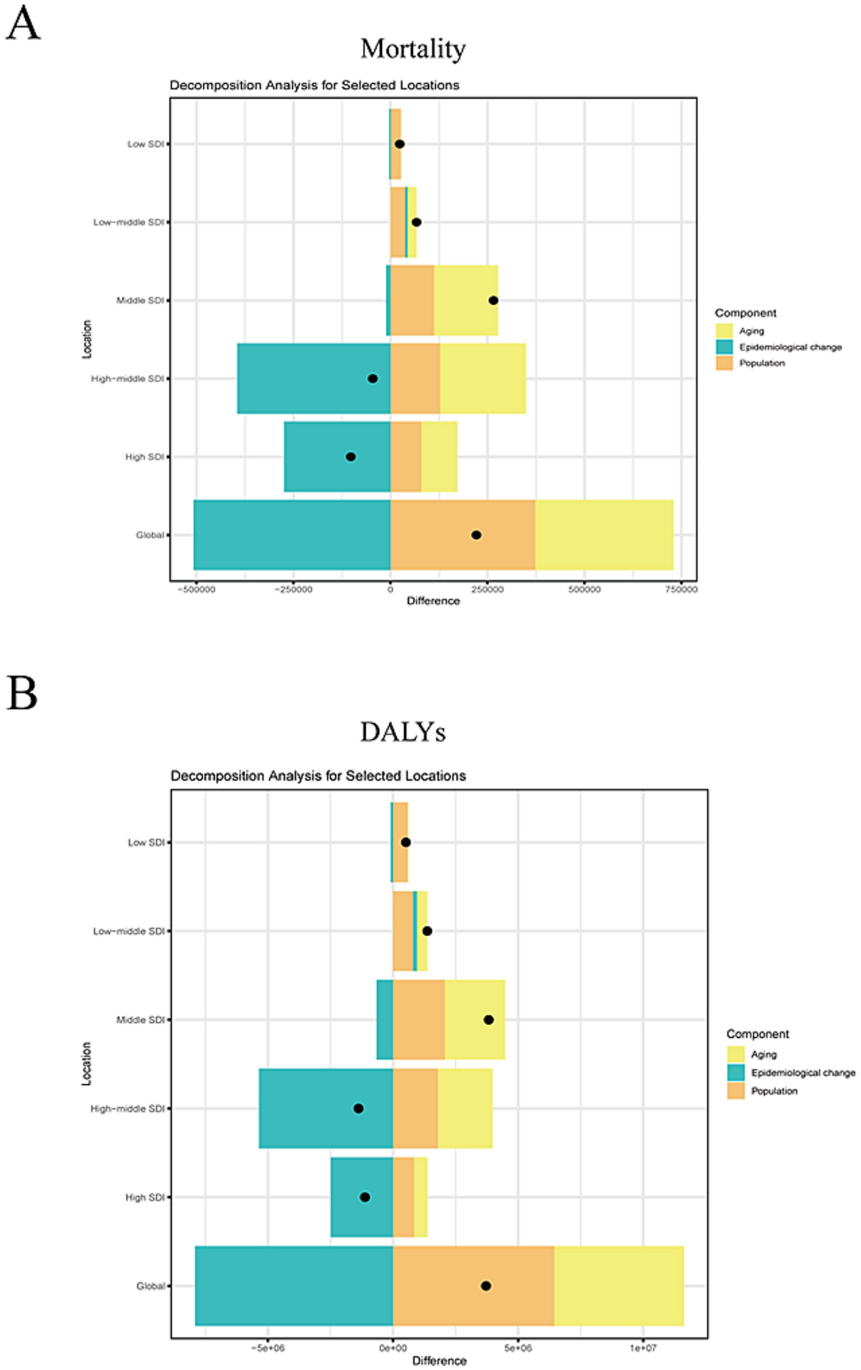
Decomposition analysis. **(A)** The effects of aging, population growth, and epidemiological changes on the mortality of high alcohol use-attributable stroke. **(B)** The effects of aging, population growth, and epidemiological changes on the DALYs of high alcohol use-attributable stroke.

This study conducts a decomposition analysis of mortality and DALYs using data from 1990 to 2021, aiming to evaluate the effects of aging, population growth, and epidemiological changes on the burden of high alcohol use-attributable stroke. Findings reveal that in global, high, high-middle and middle SDI regions, epidemiological changes are the primary drivers of decline in mortality and DALYs for high alcohol use-attributable stroke (Figures 4A,B). By contrast, aging and population growth act as contributing factors exacerbating the burden. In low-middle and low SDI regions, population growth is the primary factor exacerbating the burden of high alcohol use-attributable stroke.

### 3.6 Frontier analysis

**Figure 5:**
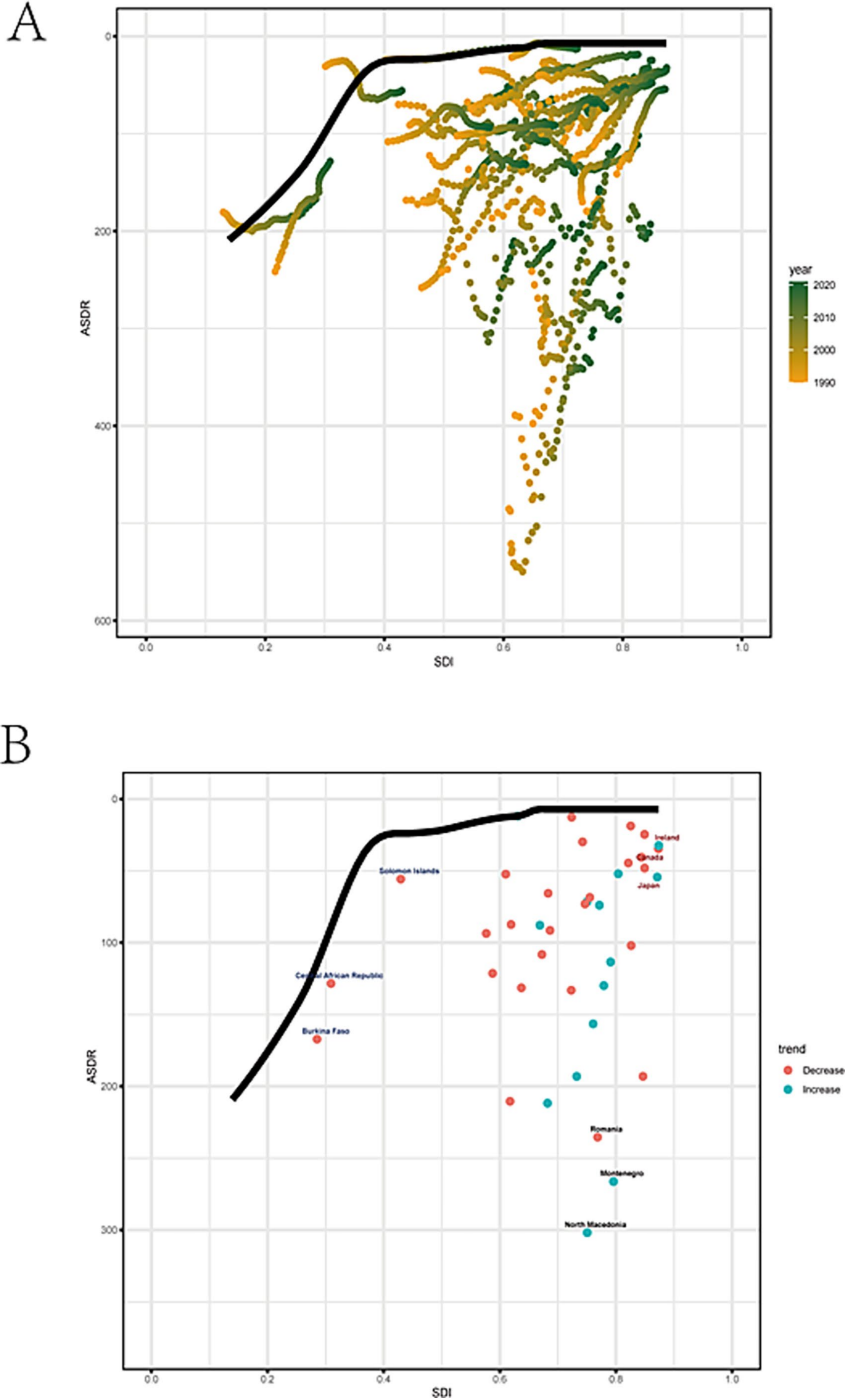
Frontier analysis. **(A)** The changes in SDI and ASDR for each country or region were presented. Boundaries were delineated with solid black lines; countries and regions were represented by points. **(B)** Black represents the top 3 countries with the largest effective difference while blue represents frontier countries with low SDI (< 0.45) and low effective difference, and red label indicates countries and territories with high SDI (> 0.85).

A frontier analysis is conducted based on ASDR and SDI to explore the improvement potential for the burden of high alcohol use-attributable stroke. Overall, as SDI increased, the effective SDI differences in ASDR tend to remain constant (Figures 5A,B). Romania, Montenegro, and North Macedonia are the 3 nations with the greatest effective differences from the minimum disease burden. This imply that these countries require enhancements to their healthcare systems. The three low SDI nations with the smallest effective differences from the minimum disease burden are the Solomon Islands, the Central African Republic, and Burkina Faso. Conversely, the three high SDI nations with the smallest effective differences are Ireland, Canada, and Japan.

### 3.7 Prediction of mortality of stroke attributable to high alcohol use in the next decade

**Figure 6:**
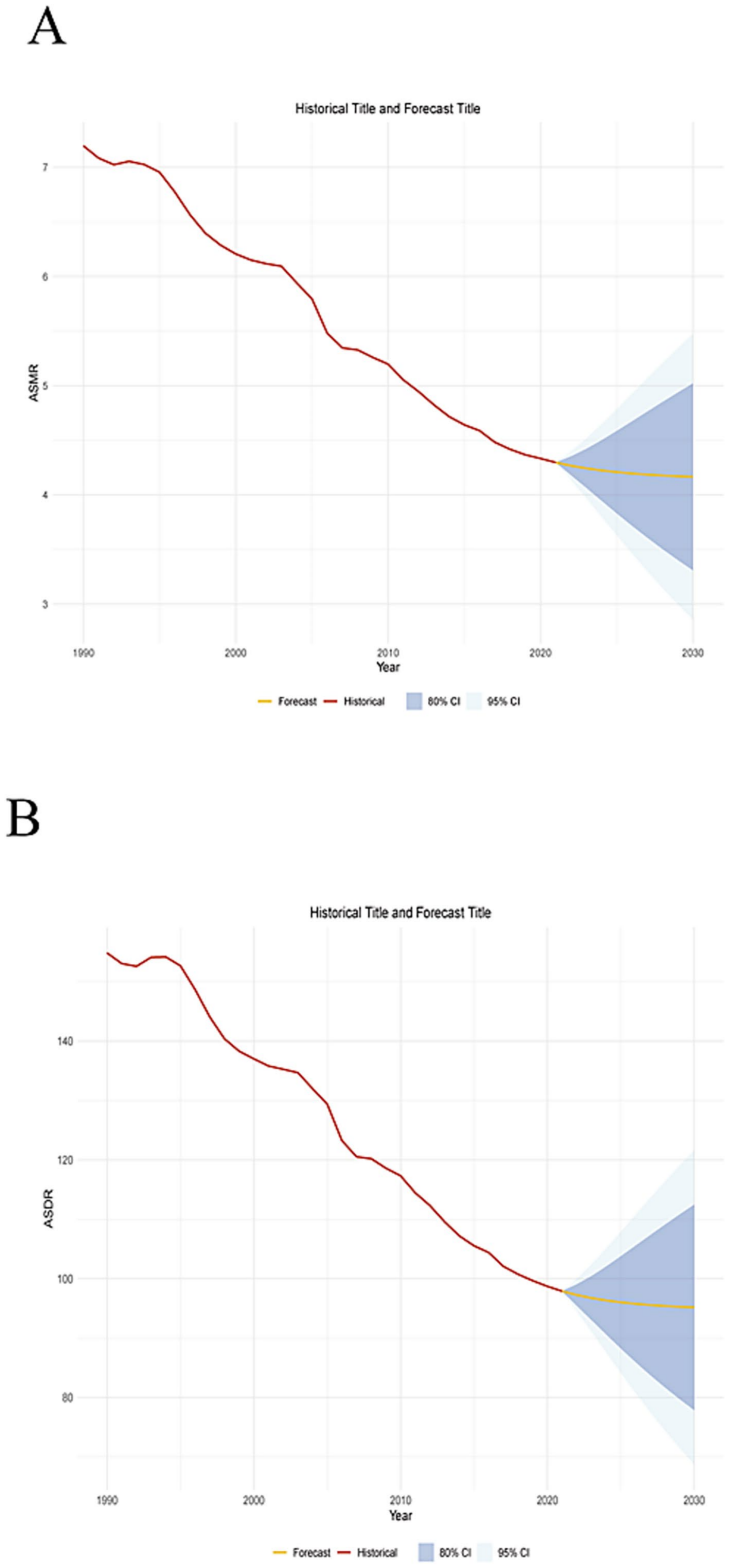
Prediction of disease burden in the next decade. **(A)** Predicted trends of ASMR. **(B)** Predicted trends of ASDR.

Using the ARIMA model, the ASMR for high alcohol use-attributable stroke is projected to decline from 4.3 per 100,000 in 2021 to 4.15 per 100,000 by 2030 (Figure 6A). Concurrently, the ASDR is forecast to decrease from 98 per 100,000 in 2021 to 95 per 100,000 over the same period (Figure 6B). However, the predicted values have a wide confidence interval, indicating a certain degree of error. Consequently, countries should further strengthen their attention to this disease, formulate relevant policies, and enhance medical security.

## 4 Discussion

In this study, we conduct a systematic evaluation of the temporal trends in the global burden of stroke attributable to high alcohol use from 1990 to 2021. Findings indicate that significant disparities persisted in the distribution of high alcohol use-attributable stroke across gender, age groups, regions, and disease subtypes. The fluctuations in this trend have been meticulously analyzed to uncover the primary factors underlying it. Eventually, projections are formulated for the trend changes in the upcoming decade, aiming to alert people to the hazards of excessive alcohol consumption.

From 1990 to 2021, the stroke death cases and DALYs attributable to high alcohol consumption increased. This trend is likely driven not only by population growth and ageing but also by social and lifestyle habits. We have outlined the potential reasons for this here. The global rise in alcohol consumption. In the developing countries such as India and Brazil, alcohol consumption has gradually become normalized in scenarios such as traditional festivals, social gatherings, and workplace events, which has led to a growing population of heavy drinkers (14). In cold regions like Eastern Europe and Russia, strong spirits (such as vodka) are heavily consumed, with annual per capita alcohol intake exceeding 15 liters on average—far surpassing the WHO’s recommended limit. In East and Southeast Asia, social drinking practices—such as South Korea’s “table culture”—have contributed to a surge in cumulative alcohol consumption among young people. Research indicates that 40% of South Korean men consume more than 105 grams of alcohol per week (15). In the context of population aging in high-income countries, individuals with alcohol use disorders are often comorbid with underlying conditions such as diabetes and hyperlipidemia, which contributes to a rise in mortality from ischemic stroke. There is an unequal distribution of global medical resources. Low-income countries lack stroke units and thrombolysis equipment, leading to delayed treatment for stroke patients with alcohol-related causes. According to WHO statistics, fewer than 15% of stroke patients in Africa reach hospitals within 3 h, and their mortality rate is 40% higher than that in developed countries (14). In addition, only a few countries include screening for alcohol use disorders in primary healthcare. Countries across the globe enforce diverse alcohol-related policies. Multinational alcohol corporations are penetrating emerging markets through low-price strategies and marketing tactics, such as promoting low-alcohol and fruit-flavored beverages. Only 34% of countries worldwide have implemented alcohol taxation, with tax rates generally lower than those on tobacco (14). However, age-standardized mortality rates (ASMR) and DALYs (ASDR) show a decline. The World Health Organization (WHO) has implemented a global strategy to reduce alcohol consumption, aiming to mitigate the substantial public health burdens associated with harmful alcohol use (16). In 2010, the WHO advocated for a national-level relative reduction of at least 10% in the harmful use of alcohol by 2025, with an additional target of at least 20% relative reduction by 2030 compared to 2010 baselines. Consequentially, global per capita alcohol consumption demonstrated a modest decline from 5.7 liters in 2010 to 5.5 liters in 2019, translating to a 4.5% relative reduction. With societal development, the dissemination of stroke symptoms by medical professionals has enabled patients to receive medical assistance within an effective time window, including anticoagulation, thrombolysis, thrombectomy and other methods. Consequently, an increasing proportion of stroke patients are now able to survive (17). Long-term rehabilitation resources and stroke care interventions can significantly improve the quality of life for patients. Additionally, several countries have implemented enhancements to their data statistics and monitoring systems to identify high-risk individuals.

Regarding gender distribution, ASMR and ASDR for stroke attributable to high alcohol consumption in males are significantly higher than those in females. The motivation for drinking is a key factor that may differ between genders and across age groups, with stress coping warranting particular scrutiny (18). Stress is strongly associated with all phases of alcohol addiction, including drinking initiation, maintenance, and relapse for both women and men. When confronted with life or work pressure, men tend to relax by consuming alcohol. However, the EAPC for females decrease more significantly than that for males. A key reason is the significantly higher alcohol consumption among men, whereas a large proportion of women drink little or abstain entirely throughout their lives. This finding is consistent with the results of the WHO survey. It was found that whether it is heavy episodic drinking (HED) or heavy continuous drinking (HCD), the number of men engaging in such behaviors is significantly higher than that of women. Over time, unhealthy drinking habits, including binge drinking and chronic alcohol use, have contributed to growing disparities that significantly increase individuals’ mortality risk from stroke. This is due to the persistent negative effects of alcohol on the heart and blood vessels (19).

Currently, the population of drinkers is becoming younger, and the number is also increasing. Alcohol consumption has emerged as a prominent means of socialization and entertainment among young people, particularly in developed countries of Europe and America (20). In addition, academic competition, employment anxiety, and family conflicts drive young people to turn to alcohol as a means of relaxation and emotional relief. Surprisingly, the earliest report age of drinking can be traced back to before the age of 10. Preliminary estimates indicate that there are currently 155 million teenagers worldwide who consume alcohol, with a 70% relapse rate within the past 30 days. The harm of alcohol stems from the long-term impact of excessive drinking on various bodily systems, particularly the cardiovascular system. Mukhopadhyay et al. (21) found that chronic alcohol consumption accelerates cardiovascular aging and further reduces the already compromised cardiac and vascular reserve capacity associated with aging. Our findings also confirm this pattern: the population of stroke attributable to high alcohol consumption with the highest number of deaths is concentrated in the 70–74 age group. Middle-aged and elderly individuals constitute the majority. Compared to younger individuals, elderly people with functional degradation are no longer effective in countering the harm caused by alcoholism. When combined with chronic conditions such as hypertension, diabetes, and hyperlipidemia, the cardiovascular system undergoes further damage, which increases the risk of adverse events (22).

Over the past 32 years, countries in high and high-middle SDI groups have shown significant declines in ASMR and ASDR, indicating that primary and secondary prevention policies formulated by the WHO have been effectively implemented across different regions (23). On one hand, in high and high-middle SDI countries, they can implement restrictive policies to control alcohol abuse, allocate sufficient funds for awareness campaigns and education, strengthen the healthcare system, and ensure medical security (24). One the other hand, most individuals in high and high-middle SDI countries have a profound understanding of the harms of alcohol abuse and can consciously abstain from drinking, reduce consumption, or even adopt a total sobriety approach. In low-middle SDI countries, owing to developmental constraints, national health promotion policies have not been prioritized as key strategies in national development agendas. Limited medical resources and permissive alcohol policies are significant contributors to the elevated mortality rate (20). Thus, changes in stroke-related ASMR and ASDR attributable to high alcohol consumption primarily depend on national-level efforts, including policy formulation, protective measures, and healthcare provision.

Ischemic stroke continues to account for the largest proportion of all new strokes, followed by intracerebral hemorrhage. The relative proportions of each pathological type vary substantially across income regions. It is well established that alcohol consumption is a risk factor for ischemic stroke. The potential mechanisms involve alcohol-induced activation of the sympathetic nervous system and the renin-angiotensin-aldosterone system, leading to vasoconstriction and coagulation abnormalities (25). Dufour et al. (26) found that heavy alcohol consumption is associated with an increased risk of hemorrhagic stroke. However, the underlying mechanisms require further investigation. One reason for the decline in stroke ASMR and ASDR in high and high-middle SDI groups is the effective implementation of primary and secondary prevention measures, which entails active promotion and robust medical support.

Limitations need to be addressed. First, we examine changes in alcohol-related stroke mortality rates, but the GBD database did not allow us to determine the specific type of alcohol consumed (such as beer, spirits, and wine). Second, the database lacks a clear definition of excessive alcohol consumption. Additionally, given the developmental disparities across regions, analysis of stroke mortality attributable to excessive alcohol consumption may be subject to bias. Third, the GBD database assesses the health burden of long-term alcohol abuse and thus cannot reflect momentary exposure. This is because some individuals drink intermittently rather than daily. Fourth, The GBD 2021 relies on modeled data rather than direct observational data, a limitation that may introduce systematic bias. Additionally, the structural characteristics of the data present challenges in controlling for confounding factors. Fifth, due to the limited capacity of low-SDI regions in data statistics and regulation, the number of cases collected may be biased. Sixth, owing to limitations in medical technology, certain countries lack the capacity to accurately identify stroke types, which gives rise to classification bias.

## 5 Conclusion

In summary, the stroke death cases attributable to high alcohol consumption continue to rise. The age-standardized mortality rate (ASMR) and age-standardized disability-adjusted life year (ASDR) for stroke attributable to high alcohol consumption decline globally between 1990 and 2021, across both sexes and in most regions, with negative estimated annual percentage changes (EAPCs). This trend is expected to persist over the next decade. Ischemic stroke and intracerebral hemorrhage attributable to high alcohol consumption both exhibits a global downward trend, as reflected by ASMR and ASDR with negative EAPCs. Therefore, relevant countries and institutions should continue to attach great importance to the impact of this disease. Stringent alcohol restriction policies should be further enforced, and a comprehensive healthcare system integrating prevention, diagnosis, and treatment should be established to alleviate the disease burden.

## Data Availability

The original contributions presented in the study are included in the article/Supplementary material, further inquiries can be directed to the corresponding authors.

## Footnotes

^1^ https://vizhub.healthdata.org/gbd-results/
