# Supplementary material for: Global burden and projections of stroke and its subtypes attributable to high alcohol use during 1990–2021: insights from the global burden of disease study 2021
Source: Front Neurol. 2025 Sep 8;16:1653790. doi: 10.3389/fneur.2025.1653790 (PMC12450713; doi:10.3389/fneur.2025.1653790)
Supplement: Supplementary file 1 [file Data_Sheet_1.docx]

Supplementary Table 1: Mortality cases, age-standardized rates of mortality (ASMR, per 100,000) of stroke attributable to high alcohol use from 1990 to 2021, and estimated annual percentage changes (EAPCs) in age-standardized rates over the same period.

|  | **1990** | | **2021** | | **1990-2021** |
| --- | --- | --- | --- | --- | --- |
|  | Death  (*10^2^; 95% UI) | ASMR  (95% UI) | Death  (*10^2^; 95% UI) | ASMR  (95% UI) | EAPC of ASMR  (95% CI) |
| Region |  |  |  |  |  |
| Andean Latin America | 5.89 [1.24-11.52] | 3.03 [0.67-5.94] | 9.46 [2.08-18.95] | 1.63 [0.35-3.27] | -1.97 [-2.29 to -1.66] |
| Australasia | 12.54 [1.32-31.08] | 5.61 [0.53-13.92] | 13.78 [2.03-28.48] | 2.17 [0.35-4.46] | -3.12 [-3.19 to -3.06] |
| Caribbean | 10.73 [2.51-21.65] | 4.23 [0.98-8.62] | 17.33 [4.05-34.64] | 3.2 [0.75-6.41] | -0.8 [-0.88 to -0.73] |
| Central Asia | 26.6 [5.38-56.04] | 5.77 [1.1-12.38] | 36.41 [7.08-78.91] | 4.74 [0.83-10.71] | -0.92 [-1.25 to -0.6] |
| Central Europe | 227.61 [33.1-467.83] | 16.26 [2.2-34.13] | 176.09 [22.38-375.3] | 7.45 [1.1-15.7] | -2.93 [-3.11 to -2.75] |
| Central Latin America | 18.89 [4.25-37.71] | 2.5 [0.53-5.15] | 29 [6.35-58.99] | 1.18 [0.26-2.41] | -2.91 [-3.15 to -2.67] |
| Central Sub-Saharan Africa | 11.81 [2.1-23.39] | 6.58 [1.2-13.3] | 24.46 [4.4-50.69] | 5.53 [1.07-11.37] | -0.29 [-0.96 to 0.39] |
| East Asia | 794.95 [179.56-1527.69] | 10.59 [2.47-20.32] | 1605.53 [388.41-3035.02] | 7.79 [1.83-14.61] | -0.92 [-1.04 to -0.79] |
| Eastern Europe | 308.75 [30.6-711.66] | 11.39 [1.06-27.14] | 234.36 [23.12-570.99] | 6.63 [0.77-16.04] | -2.6 [-3.29 to -1.91] |
| Eastern Sub-Saharan Africa | 39.2 [6.08-80.86] | 5.94 [1.07-11.95] | 72.58 [15.55-143.43] | 4.93 [1.11-9.54] | -0.83 [-1.04 to -0.62] |
| High-income Asia Pacific | 148.23 [28.35-285.43] | 8.1 [1.53-15.76] | 126.3 [24.68-253.02] | 2.16 [0.44-4.19] | -4.63 [-4.81 to -4.45] |
| High-income North America | 72.32 [10.43-193.66] | 2 [0.31-5.27] | 135.79 [22.86-298.18] | 1.92 [0.35-4.13] | -0.25 [-0.39 to -0.1] |
| North Africa and Middle East | 11.75 [2.47-24.87] | 0.76 [0.14-1.64] | 14.71 [2.47-33.36] | 0.37 [0.05-0.85] | -2.58 [-2.67 to -2.49] |
| Oceania | 0.76 [0.1-1.72] | 2.69 [0.39-6.09] | 1.38 [0.18-3.1] | 1.92 [0.3-4.25] | -0.87 [-1.22 to -0.52] |
| South Asia | 80.61 [13.71-174.24] | 1.48 [0.25-3.26] | 261.1 [62.98-542.23] | 1.84 [0.43-3.82] | 0.92 [0.75 to 1.08] |
| Southeast Asia | 57.34 [12.28-110.58] | 2.4 [0.55-4.57] | 314.54 [76.72-581.35] | 5.08 [1.25-9.3] | 2.86 [2.54 to 3.17] |
| Southern Latin America | 45.69 [10.91-86.84] | 10.38 [2.3-19.87] | 28.3 [5.7-55.76] | 3.13 [0.63-6.13] | -3.57 [-3.73 to -3.4] |
| Southern Sub-Saharan Africa | 14 [3.33-27.32] | 5.71 [1.28-11.11] | 29.41 [7.2-57.07] | 5.73 [1.39-11.31] | -0.12 [-0.57 to 0.33] |
| Tropical Latin America | 53.51 [11.79-102.7] | 6.24 [1.22-12.2] | 62.57 [12.77-123.88] | 2.48 [0.49-4.93] | -3.02 [-3.16 to -2.88] |
| Western Europe | 539.04 [60.82-1140.16] | 8.9 [1.08-18.76] | 306.68 [50.02-628.06] | 2.59 [0.47-5.2] | -4.12 [-4.25 to -3.99] |
| Western Sub-Saharan Africa | 56.01 [12.92-108.97] | 7.29 [1.62-14.51] | 107.44 [23.65-206.84] | 6.48 [1.28-12.79] | -0.53 [-0.67 to -0.39] |
| Southeast Asia, East Asia, and Oceania | 853.05 [191.57-1636.44] | 8.58 [2-16.39] | 1921.45 [472.42-3563.7] | 7.24 [1.76-13.43] | -0.42 [-0.55 to -0.29] |
| Central Europe, Eastern Europe, and Central Asia | 562.96 [69.92-1231.58] | 12.38 [1.44-27.73] | 446.86 [50.64-1048.52] | 6.79 [0.82-15.88] | -2.6 [-3.08 to -2.12] |
| High-income | 817.82 [114.61-1706] | 6.71 [0.94-13.97] | 610.85  [110.42-1255.65] | 2.32 [0.43-4.68] | -3.59 [-3.73 to -3.45] |
| Latin America and Caribbean | 89.03 [20.49-172.17] | 4.27 [0.9-8.47] | 118.35 [25.46-232.89] | 1.94 [0.4-3.85] | -2.69 [-2.76 to -2.63] |
| Sub-Saharan Africa | 121.02 [26.27-233.19] | 6.59 [1.49-12.78] | 233.89 [55.66-447.32] | 5.75 [1.35-11.06] | -0.58 [-0.65 to -0.52] |
