# Supplementary material for: Global burden and projections of stroke and its subtypes attributable to high alcohol use during 1990–2021: insights from the global burden of disease study 2021
Source: Front Neurol. 2025 Sep 8;16:1653790. doi: 10.3389/fneur.2025.1653790 (PMC12450713; doi:10.3389/fneur.2025.1653790)
Supplement: Supplementary file 2 [file Data_Sheet_2.docx]

Supplementary Table 2: DALYs cases, age-standardized rates of DALYs (ASDR, per 100,000) of stroke attributable to high alcohol use from 1990 to 2021, and estimated annual percentage changes (EAPCs) in age-standardized rates over the same period.

|  | **1990** | | **2021** | | **1990-2021** |
| --- | --- | --- | --- | --- | --- |
|  | DALY  (*10^2^; 95% UI) | ASDR  (95% UI) | DALY  (*10^2^; 95% UI) | ASDR  (95% UI) | EAPC of ASDR (95% CI) |
| Region |  |  |  |  |  |
| Andean Latin America | 154.43 [26.29-323.69] | 73.93 [14.09-145.26] | 239.43 [50.11-470.61] | 39.85 [8.71-77.75] | -1.99 [-2.31 to -1.66] |
| Australasia | 234.02 [33.32-554.8] | 101 [14.31-238.12] | 240.74 [36.31-497.9] | 41.63 [6.73-84.85] | -2.93 [-3.01 to -2.85] |
| Caribbean | 282.98 [59.75-565.37] | 107.37 [23.34-213.11] | 435.4 [96.35-884.19] | 81.18 [17.86-164.74] | -0.8 [-0.91 to -0.7] |
| Central Asia | 779.96 [181.65-1561.09] | 160 [35.99-326.55] | 1036.44 [223.86-2117.38] | 120.51 [24.14-251.92] | -1.34 [-1.7 to -0.97] |
| Central Europe | 4953.36 [895.7-9802.42] | 335.97 [59.13-669.09] | 3470.95 [575.79-7045.2] | 155.5 [25.41-311.62] | -2.94 [-3.13 to -2.75] |
| Central Latin America | 486.3 [107.59-941.59] | 57.83 [13.15-111.36] | 743.97 [174.72-1478.97] | 29.17 [6.69-57.8] | -2.76 [-3.02 to -2.49] |
| Central Sub-Saharan Africa | 305.92 [46.27-636.29] | 144.27 [25.44-290.94] | 654.06 [103.27-1435.86] | 122.94 [21.82-253.35] | -0.21 [-0.88 to 0.46] |
| East Asia | 20859.66 [4345.29-40551.85] | 239.32 [51.99-459.13] | 36922.04 [9243.11-69496.29] | 170.56 [42.18-320.85] | -1.01 [-1.11 to -0.9] |
| Eastern Europe | 7598.15 [1000.51-16529.54] | 269.63 [35.17-595.23] | 5824.91 [856.14-13104.93] | 170.28 [26.94-379.42] | -2.34 [-3.04 to -1.63] |
| Eastern Sub-Saharan Africa | 1064.15 [124.88-2225.45] | 140.11 [21.86-288.47] | 1971.45 [376.86-3930.04] | 114.78 [24.6-224.38] | -0.91 [-1.13 to -0.69] |
| High-income Asia Pacific | 3403.38 [700.94-6265.66] | 172.46 [34.61-319.25] | 2504.1 [452.69-4914.09] | 54.96 [10.14-104.91] | -4.03 [-4.21 to -3.85] |
| High-income North America | 1674.46 [298.13-4164.99] | 48.37 [9.21-118.57] | 3026.3 [557.68-6353.42] | 47.33 [9.23-98.08] | -0.15 [-0.24 to -0.05] |
| North Africa and Middle East | 351.17 [75.24-734.54] | 19.11 [4.15-40.57] | 412.33 [77.71-895.06] | 8.65 [1.51-19.41] | -2.83 [-2.91 to -2.75] |
| Oceania | 24.69 [2.75-55.78] | 72.3 [10.01-161.07] | 44.5 [5.53-100.85] | 51.18 [7.09-113.92] | -0.9 [-1.24 to -0.56] |
| South Asia | 2326.84 [369.28-4955.73] | 38.01 [6.43-81.45] | 7088.85 [1721.06-14427.98] | 46.1 [11.19-93.57] | 0.83 [0.67 to 0.99] |
| Southeast Asia | 1676.13 [307.52-3325.68] | 61.86 [12.27-120.77] | 8753.36 [2113.43-16267.37] | 126.87 [31.1-236.57] | 2.73 [2.44 to 3.02] |
| Southern Latin America | 1085.24 [280.92-1988.84] | 236.18 [60.09-432.76] | 618.5 [134.82-1185.67] | 70.72 [15.93-135.18] | -3.73 [-3.88 to -3.57] |
| Southern Sub-Saharan Africa | 381.1 [79.94-751.63] | 138.09 [30.28-268.62] | 762.87 [168.94-1467.01] | 131.41 [31.44-253.37] | -0.31 [-0.72 to 0.09] |
| Tropical Latin America | 1472.68 [347.75-2767.03] | 152.88 [36.15-289.35] | 1508.47 [337.19-2851.22] | 58.04 [12.81-109.68] | -3.28 [-3.4 to -3.16] |
| Western Europe | 9486.12 [1312.81-19494.51] | 159.91 [24.66-326.36] | 5266.19 [913.01-10666.16] | 51.65 [9.34-102.07] | -3.8 [-3.94 to -3.66] |
| Western Sub-Saharan Africa | 1431.67 [321.08-2819.08] | 164.15 [37.97-320.15] | 2847.91 [666.22-5344.82] | 143.94 [31.56-277.83] | -0.58 [-0.74 to -0.42] |
| Southeast Asia, East Asia, and Oceania | 22560.48 [4697.22-44119.9] | 197.68 [42.99-380.19] | 45719.89 [11953.04-84514.67] | 161.34 [41.36-299.87] | -0.52 [-0.63 to -0.4] |
| Central Europe, Eastern Europe, and Central Asia | 13331.48 [2086.5-27483.01] | 280.24 [43.02-585.44] | 10332.3 [1604.54-22551.97] | 161.07 [26.37-345.72] | -2.48 [-2.99 to -1.97] |
| High-income | 15883.23 [2679.67-32064.74] | 132.13 [23.14-264.65] | 11655.82 [2129.9-23586.9] | 51.67 [9.61-101.52] | -3.2 [-3.34 to -3.06] |
| Latin America and Caribbean | 2396.39 [539.38-4571.25] | 104.12 [24.6-199.27] | 2927.25 [681.24-5607.62] | 46.54 [10.77-89.65] | -2.82 [-2.91 to -2.74] |
| Sub-Saharan Africa | 3182.84 [612.31-6304.44] | 150.68 [32.1-293.41] | 6236.28 [1357.45-11883.69] | 129.85 [30.89-249.41] | -0.64 [-0.7 to -0.57] |
