# Supplementary material for: Global burden and projections of stroke and its subtypes attributable to high alcohol use during 1990–2021: insights from the global burden of disease study 2021
Source: Front Neurol. 2025 Sep 8;16:1653790. doi: 10.3389/fneur.2025.1653790 (PMC12450713; doi:10.3389/fneur.2025.1653790)
Supplement: Supplementary file 3 [file Data_Sheet_3.docx]

Supplementary Table 3: Mortality cases, age-standardized rates of mortality (ASMR, per 100,000) of stroke subtypes attributable to high alcohol use from 1990 to 2021, and estimated annual percentage changes (EAPCs) in age-standardized rates over the same period.

|  | **1990** | | **2021** | | **1990-2021** |
| --- | --- | --- | --- | --- | --- |
|  | Death  (*10^2^ ; 95% UI) | ASMR  (95% UI) | Death  (*10^2^; 95% UI) | ASMR  (95% UI) | EAPC of ASMR  (95% CI) |
| Ischemic stroke |  |  |  |  |  |
| Region |  |  |  |  |  |
| Andean Latin America | 2.23 [0.34-5.48] | 1.25 [0.18-3.08] | 4.32 [0.58-11.09] | 0.77 [0.1-1.98] | -1.51 [-1.82 to -1.2] |
| Australasia | 9.22 [0.67-26.57] | 4.2 [0.29-12.12] | 9.48 [0.91-23.29] | 1.44 [0.14-3.53] | -3.56 [-3.64 to -3.48] |
| Caribbean | 4.29 [0.49-11.25] | 1.78 [0.2-4.68] | 7.98 [1.01-21.16] | 1.47 [0.19-3.89] | -0.47 [-0.56 to -0.38] |
| Central Asia | 12.27 [1.26-35.09] | 2.78 [0.27-8.11] | 17.94 [1.88-51.11] | 2.52 [0.25-7.43] | -0.64 [-0.9 to -0.39] |
| Central Europe | 153.07 [13.01-363.31] | 11.27 [0.86-26.96] | 130.56 [13.23-316.11] | 5.39 [0.56-13.07] | -2.68 [-2.83 to -2.53] |
| Central Latin America | 9.1 [1.2-23.82] | 1.32 [0.17-3.48] | 13.72 [1.98-36.86] | 0.58 [0.08-1.57] | -2.98 [-3.25 to -2.71] |
| Central Sub-Saharan Africa | 3.51 [0.54-9.94] | 2.51 [0.35-6.99] | 7.91 [1.04-22.04] | 2.27 [0.28-6.29] | -0.1 [-0.79 to 0.6] |
| East Asia | 240.8 [44.16-588.45] | 3.51 [0.64-8.57] | 715.9 [107.57-1741.43] | 3.56 [0.49-8.66] | 0.2 [0.06 to 0.35] |
| Eastern Europe | 231.72 [15.77-607.48] | 8.67 [0.56-23.46] | 171.61 [13.48-488.86] | 4.75 [0.38-13.56] | -2.77 [-3.43 to -2.1] |
| Eastern Sub-Saharan Africa | 7.92 [1.17-21.18] | 1.54 [0.24-4.03] | 20.95 [3.57-54.97] | 1.74 [0.27-4.56] | 0.26 [0.12 to 0.4] |
| High-income Asia Pacific | 90.03 [10.91-205.82] | 5.16 [0.6-11.81] | 81.41 [11.71-193.87] | 1.22 [0.19-2.88] | -5.13 [-5.34 to -4.92] |
| High-income North America | 47.94 [4.08-154.63] | 1.29 [0.11-4.13] | 84.01 [8.9-216.7] | 1.13 [0.12-2.9] | -0.69 [-0.95 to -0.43] |
| North Africa and Middle East | 5.68 [0.6-15.56] | 0.41 [0.04-1.11] | 9.18 [0.82-26.27] | 0.25 [0.02-0.72] | -1.79 [-1.85 to -1.73] |
| Oceania | 0.1 [0.01-0.29] | 0.5 [0.06-1.43] | 0.21 [0.03-0.63] | 0.41 [0.05-1.22] | -0.63 [-0.91 to -0.36] |
| South Asia | 25.17 [2.85-78.65] | 0.54 [0.06-1.68] | 100.32 [12.34-288.01] | 0.78 [0.09-2.25] | 1.31 [1.17 to 1.44] |
| Southeast Asia | 17.81 [2.98-44.47] | 0.88 [0.13-2.2] | 113.79 [17.84-267.75] | 2.09 [0.3-4.89] | 3.28 [2.95 to 3.6] |
| Southern Latin America | 23.02 [2.46-54.79] | 5.47 [0.58-13.17] | 15.92 [2.07-39.04] | 1.72 [0.23-4.21] | -3.29 [-3.49 to -3.09] |
| Southern Sub-Saharan Africa | 5.65 [0.94-14.4] | 2.61 [0.43-6.64] | 12.66 [2.04-31.54] | 2.81 [0.42-7.12] | 0.15 [-0.31 to 0.62] |
| Tropical Latin America | 26.46 [3.23-65.03] | 3.46 [0.38-8.48] | 35.29 [4.86-86.13] | 1.43 [0.19-3.5] | -2.77 [-2.89 to -2.65] |
| Western Europe | 410.38 [34.73-969.71] | 6.71 [0.56-15.9] | 212.63 [24.45-493.41] | 1.7 [0.21-3.94] | -4.6 [-4.76 to -4.43] |
| Western Sub-Saharan Africa | 23.73 [4-61.91] | 3.49 [0.58-9.17] | 53.7 [8.92-130.66] | 3.7 [0.58-9.11] | 0.13 [-0.04 to 0.3] |
| Southeast Asia, East Asia, and Oceania | 258.71 [46.08-630.64] | 2.85 [0.5-6.94] | 829.9 [125.45-2031.22] | 3.26 [0.45-7.96] | 0.63 [0.49 to 0.78] |
| Central Europe, Eastern Europe, and Central Asia | 397.06 [31.58-992.99] | 8.91 [0.68-22.59] | 320.12 [27.51-856.18] | 4.79 [0.41-12.81] | -2.63 [-3.1 to -2.16] |
| High-income | 580.59 [56.43-1382.77] | 4.73 [0.45-11.3] | 403.44 [49.13-957.34] | 1.43 [0.19-3.38] | -4.08 [-4.25 to -3.9] |
| Latin America and Caribbean | 42.07 [5.54-105.66] | 2.22 [0.27-5.57] | 61.3 [8.65-153.75] | 1.03 [0.14-2.6] | -2.5 [-2.55 to -2.45] |
| Sub-Saharan Africa | 40.81 [6.97-105.75] | 2.65 [0.44-6.91] | 95.22 [16.53-242.65] | 2.76 [0.45-6.97] | 0.05 [-0.07 to 0.16] |
| Intracerebral hemorrhage |  |  |  |  |  |
| Region |  |  |  |  |  |
| Andean Latin America | 3.66 [0.15-8.08] | 1.78 [0.08-3.95] | 5.14 [0.1-11.05] | 0.86 [0.02-1.84] | -2.35 [-2.67 to -2.02] |
| Australasia | 3.32 [0.1-7.65] | 1.41 [0.05-3.24] | 4.3 [0.14-8.75] | 0.73 [0.02-1.49] | -2.03 [-2.11 to -1.95] |
| Caribbean | 6.44 [0.15-14.16] | 2.45 [0.06-5.44] | 9.35 [0.18-20.26] | 1.74 [0.03-3.76] | -1.07 [-1.19 to -0.95] |
| Central Asia | 14.33 [0.52-31.29] | 2.99 [0.11-6.65] | 18.47 [0.5-40.73] | 2.22 [0.07-4.98] | -1.21 [-1.63 to -0.79] |
| Central Europe | 74.54 [3.18-154.83] | 4.99 [0.21-10.44] | 45.53 [1.86-94.51] | 2.06 [0.08-4.24] | -3.51 [-3.78 to -3.24] |
| Central Latin America | 9.79 [0.26-21.29] | 1.18 [0.03-2.56] | 15.28 [0.37-33.55] | 0.6 [0.01-1.32] | -2.84 [-3.07 to -2.61] |
| Central Sub-Saharan Africa | 8.3 [0.1-19.53] | 4.07 [0.05-9.47] | 16.54 [0.24-38.91] | 3.26 [0.08-7.54] | -0.41 [-1.07 to 0.26] |
| East Asia | 554.15 [7.51-1167.76] | 7.09 [0.09-14.92] | 889.63 [10.81-1873.93] | 4.23 [0.05-8.93] | -1.63 [-1.78 to -1.49] |
| Eastern Europe | 77.03 [2.72-167.79] | 2.71 [0.09-5.98] | 62.75 [2.01-137.77] | 1.88 [0.05-4.07] | -2.14 [-2.9 to -1.37] |
| Eastern Sub-Saharan Africa | 31.28 [0.77-70.31] | 4.4 [0.07-9.77] | 51.64 [1.6-112.58] | 3.19 [0.11-6.91] | -1.31 [-1.55 to -1.07] |
| High-income Asia Pacific | 58.2 [1.11-120.44] | 2.94 [0.06-6.11] | 44.9 [0.75-95.04] | 0.93 [0.02-1.96] | -3.84 [-4.01 to -3.68] |
| High-income North America | 24.38 [0.98-57.55] | 0.71 [0.03-1.65] | 51.78 [1.83-116.46] | 0.79 [0.03-1.74] | 0.43 [0.27 to 0.6] |
| North Africa and Middle East | 6.08 [0.13-14.37] | 0.34 [0.01-0.82] | 5.52 [0.19-13.19] | 0.12 [0-0.29] | -3.81 [-3.96 to -3.66] |
| Oceania | 0.66 [0.02-1.59] | 2.19 [0.08-5.26] | 1.16 [0.04-2.88] | 1.52 [0.06-3.7] | -0.93 [-1.3 to -0.56] |
| South Asia | 55.44 [2.61-131.25] | 0.93 [0.04-2.2] | 160.78 [4.92-369.78] | 1.06 [0.03-2.46] | 0.66 [0.47 to 0.84] |
| Southeast Asia | 39.54 [1.06-84.12] | 1.52 [0.05-3.25] | 200.75 [6.57-401.08] | 2.99 [0.1-6] | 2.59 [2.27 to 2.9] |
| Southern Latin America | 22.68 [0.55-46.48] | 4.9 [0.12-10.05] | 12.37 [0.29-25.84] | 1.41 [0.03-2.95] | -3.88 [-4.03 to -3.74] |
| Southern Sub-Saharan Africa | 8.35 [0.25-18.14] | 3.1 [0.1-6.68] | 16.75 [0.55-35.14] | 2.91 [0.1-6.11] | -0.37 [-0.81 to 0.07] |
| Tropical Latin America | 27.06 [0.9-56.07] | 2.77 [0.1-5.8] | 27.28 [0.69-56.33] | 1.04 [0.03-2.16] | -3.35 [-3.54 to -3.16] |
| Western Europe | 128.66 [5.36-269.97] | 2.19 [0.09-4.57] | 94.05 [2.93-195.63] | 0.89 [0.03-1.83] | -3.01 [-3.1 to -2.91] |
| Western Sub-Saharan Africa | 32.28 [1.13-73.75] | 3.81 [0.14-8.73] | 53.74 [1.9-114.71] | 2.77 [0.1-5.95] | -1.25 [-1.39 to -1.11] |
| Southeast Asia, East Asia, and Oceania | 594.34 [8.2-1250.07] | 5.73 [0.08-12.09] | 1091.54 [19.05-2252.86] | 3.99 [0.07-8.23] | -1.09 [-1.23 to -0.95] |
| Central Europe, Eastern Europe, and Central Asia | 165.9 [6.67-352.19] | 3.46 [0.14-7.43] | 126.75 [4.93-268.47] | 2 [0.07-4.2] | -2.53 [-3.04 to -2] |
| High-income | 237.23 [7.75-494.45] | 1.98 [0.06-4.12] | 207.4 [6.39-443.95] | 0.89 [0.03-1.89] | -2.65 [-2.72 to -2.57] |
| Latin America and Caribbean | 46.96 [1.55-100.21] | 2.05 [0.07-4.4] | 57.05 [1.35-124.05] | 0.91 [0.02-1.97] | -2.9 [-3 to -2.81] |
| Sub-Saharan Africa | 80.22 [2.09-179.95] | 3.94 [0.12-8.79] | 138.67 [5.02-299.41] | 2.99 [0.12-6.4] | -1.08 [-1.17 to -1] |
