# Supplementary material for: Global burden and projections of stroke and its subtypes attributable to high alcohol use during 1990–2021: insights from the global burden of disease study 2021
Source: Front Neurol. 2025 Sep 8;16:1653790. doi: 10.3389/fneur.2025.1653790 (PMC12450713; doi:10.3389/fneur.2025.1653790)
Supplement: Supplementary file 4 [file Data_Sheet_4.docx]

Supplementary Table 4: DALYs cases, age-standardized rates of DALYs (ASDR, per 100,000) of stroke subtypes attributable to high alcohol use from 1990 to 2021, and estimated annual percentage changes (EAPCs) in age-standardized rates over the same period.

|  | **1990** | | **2021** | | **1990-2021** |
| --- | --- | --- | --- | --- | --- |
|  | DALY  (*10^2^; 95% UI) | ASDR  (95% UI) | DALY  (*10^2^; 95% UI) | ASDR  (95% UI) | EAPC of ASDR (95% CI) |
| Ischemic stroke |  |  |  |  |  |
| Region |  |  |  |  |  |
| Andean Latin America | 47.99 [7.11-119.75] | 25.13 [3.97-61.87] | 92.75 [14.5-234.97] | 15.99 [2.47-40.57] | -1.4 [-1.71 to -1.09] |
| Australasia | 158.81 [15.08-447.91] | 68.83 [6.43-192.21] | 161.57 [20.53-389.06] | 26.91 [3.56-64.99] | -3.16 [-3.24 to -3.07] |
| Caribbean | 92.6 [12.97-239.28] | 36.35 [5.03-93.85] | 165.27 [22.53-431.02] | 30.64 [4.19-79.83] | -0.39 [-0.47 to -0.31] |
| Central Asia | 336.96 [45.25-893.34] | 71.44 [9.04-193.3] | 480.51 [63.99-1297.32] | 59.24 [6.99-165.05] | -1.05 [-1.36 to -0.75] |
| Central Europe | 3008.09 [332.1-7046.74] | 207.37 [21.93-485.97] | 2422.31 [299.12-5834] | 104.21 [13.38-251.57] | -2.52 [-2.66 to -2.38] |
| Central Latin America | 200.13 [29.16-527.99] | 26.04 [3.72-67.72] | 303.32 [49.49-782.81] | 12.31 [1.97-31.86] | -2.8 [-3.1 to -2.5] |
| Central Sub-Saharan Africa | 82.21 [12.96-231.4] | 47.55 [7.25-132.7] | 187.77 [27.12-530.13] | 43.73 [5.76-121.4] | 0.01 [-0.68 to 0.69] |
| East Asia | 5952.94 [1123.03-14550.43] | 73.44 [13.7-178.55] | 15673.96 [2753.09-38566.52] | 72.79 [12.22-179.19] | 0.13 [-0.01 to 0.26] |
| Eastern Europe | 5289.25 [428.04-13354.22] | 187.94 [14.97-480.84] | 3926.66 [363.19-10529.31] | 110.51 [10.58-298.53] | -2.51 [-3.17 to -1.85] |
| Eastern Sub-Saharan Africa | 189.71 [29.97-509.92] | 30.46 [4.53-79.37] | 505.1 [97.59-1318.3] | 35.19 [6.39-91.25] | 0.34 [0.2 to 0.48] |
| High-income Asia Pacific | 1800.07 [272.16-4161.99] | 94.57 [13.51-218.68] | 1525.77 [240.95-3473.02] | 29.45 [5.06-68.63] | -4.23 [-4.44 to -4.03] |
| High-income North America | 1017.85 [114.77-3117.75] | 28.29 [3.33-85.65] | 1799.84 [239.84-4671.09] | 26.85 [3.77-69.83] | -0.37 [-0.55 to -0.19] |
| North Africa and Middle East | 149.02 [17.87-412.9] | 9.04 [1.02-24.84] | 237.25 [27.56-683.16] | 5.33 [0.55-15.25] | -1.87 [-1.94 to -1.81] |
| Oceania | 3.11 [0.43-9.08] | 11.57 [1.49-33.6] | 6.46 [0.92-18.66] | 9.43 [1.28-28] | -0.57 [-0.82 to -0.31] |
| South Asia | 647.24 [81.25-2056.61] | 12 [1.43-37.17] | 2382.7 [332.5-6959.66] | 16.68 [2.19-48.5] | 1.15 [1.03 to 1.27] |
| Southeast Asia | 450.9 [79.6-1116.68] | 19.11 [3.29-47.8] | 2711.64 [481.41-6339.64] | 43.63 [7.26-101.91] | 3.08 [2.8 to 3.36] |
| Southern Latin America | 452.49 [60.8-1062.52] | 101.14 [13.05-239] | 309.86 [47.77-749.7] | 34.36 [5.39-83.12] | -3.17 [-3.35 to -2.99] |
| Southern Sub-Saharan Africa | 130.98 [24.24-336.38] | 53.47 [9.49-137.94] | 285.18 [50.42-720.56] | 55.16 [9.46-138.44] | -0.02 [-0.42 to 0.39] |
| Tropical Latin America | 598.05 [83.87-1482.21] | 69.25 [9.31-170.14] | 723.4 [113.54-1744.45] | 28.49 [4.41-68.84] | -2.92 [-3 to -2.84] |
| Western Europe | 6627.95 [702.3-15561.89] | 108.46 [11.72-254.96] | 3553.52 [498.8-8222.63] | 32.77 [4.95-76.93] | -4.01 [-4.19 to -3.84] |
| Western Sub-Saharan Africa | 533.7 [94.59-1379.41] | 67.92 [11.82-175.98] | 1250.35 [217.61-3019.79] | 72.27 [12.32-175.56] | 0.13 [-0.05 to 0.32] |
| Southeast Asia, East Asia, and Oceania | 6406.96 [1195.37-15701.27] | 60.61 [11.12-147.31] | 18392.06 [3200.19-45601.75] | 66.39 [11.05-164.87] | 0.49 [0.35 to 0.63] |
| Central Europe, Eastern Europe, and Central Asia | 8634.3 [794.67-21188.66] | 182.92 [16.31-451.85] | 6829.47 [701.68-17500.01] | 103.52 [10.99-264.93] | -2.48 [-2.95 to -2] |
| High-income | 10057.18 [1102.37-24022.62] | 81.51 [9.04-194.89] | 7350.55 [1049.2-17417.09] | 30.25 [4.63-72.12] | -3.41 [-3.59 to -3.23] |
| Latin America and Caribbean | 938.76 [134.53-2362.86] | 44.79 [6.24-112.04] | 1284.74 [204.81-3212.42] | 20.99 [3.29-52.54] | -2.56 [-2.65 to -2.48] |
| Sub-Saharan Africa | 936.61 [164.54-2438.91] | 51.38 [8.59-132.4] | 2228.41 [414.73-5559.78] | 54.11 [9.77-134.95] | 0.09 [-0.02 to 0.2] |
| Intracerebral hemorrhage |  |  |  |  |  |
| Region |  |  |  |  |  |
| Andean Latin America | 106.44 [2.07-248.42] | 48.81 [1.55-109.73] | 146.68 [2.2-327.41] | 23.86 [0.39-52.62] | -2.33 [-2.67 to -2] |
| Australasia | 75.21 [3.29-166.47] | 32.17 [1.52-70.68] | 79.18 [2.1-160.47] | 14.71 [0.38-29.57] | -2.48 [-2.57 to -2.39] |
| Caribbean | 190.39 [3.56-416.09] | 71.01 [1.46-153.88] | 270.13 [4.15-581.88] | 50.54 [0.76-108.61] | -1.04 [-1.18 to -0.89] |
| Central Asia | 443.01 [13.37-964.62] | 88.56 [2.89-191.63] | 555.94 [13.93-1194.38] | 61.28 [1.63-133.79] | -1.58 [-2.01 to -1.15] |
| Central Europe | 1945.27 [68.78-3949.71] | 128.6 [4.63-261.49] | 1048.64 [38.62-2110.39] | 51.3 [1.91-102.25] | -3.66 [-3.94 to -3.37] |
| Central Latin America | 286.17 [6.53-625.32] | 31.79 [0.83-68.64] | 440.64 [9.37-947.51] | 16.85 [0.36-36.41] | -2.72 [-2.98 to -2.46] |
| Central Sub-Saharan Africa | 223.71 [2.67-536.32] | 96.73 [1.21-229] | 466.29 [5.58-1102.34] | 79.21 [1.17-185.51] | -0.32 [-0.98 to 0.34] |
| East Asia | 14906.72 [211.45-31242.94] | 165.88 [2.32-348.11] | 21248.08 [274.93-44677.89] | 97.78 [1.35-205.58] | -1.65 [-1.77 to -1.53] |
| Eastern Europe | 2308.9 [77.66-4819.92] | 81.69 [2.66-171.29] | 1898.25 [55.03-3905.84] | 59.77 [1.47-121.19] | -1.99 [-2.78 to -1.18] |
| Eastern Sub-Saharan Africa | 874.43 [16.47-2065.54] | 109.65 [2.57-247.53] | 1466.34 [33.9-3311.35] | 79.59 [2.44-173.17] | -1.35 [-1.6 to -1.11] |
| High-income Asia Pacific | 1603.31 [36.77-3251.75] | 77.9 [1.76-158.09] | 978.32 [17.69-2021.93] | 25.51 [0.46-52.56] | -3.78 [-3.95 to -3.6] |
| High-income North America | 656.61 [23.55-1473.38] | 20.08 [0.69-44.46] | 1226.46 [37.57-2653.92] | 20.49 [0.62-43.89] | 0.14 [0.03 to 0.25] |
| North Africa and Middle East | 202.14 [3.9-474.02] | 10.07 [0.21-23.55] | 175.08 [5.5-407.63] | 3.31 [0.11-7.84] | -3.99 [-4.12 to -3.86] |
| Oceania | 21.58 [0.81-51.64] | 60.73 [2.35-146.18] | 38.04 [0.99-93.26] | 41.75 [1.29-103.01] | -0.97 [-1.32 to -0.61] |
| South Asia | 1679.6 [82.9-4031.21] | 26 [1.25-61.71] | 4706.15 [142.3-10771.81] | 29.43 [0.9-67.59] | 0.66 [0.48 to 0.85] |
| Southeast Asia | 1225.23 [20.82-2624.59] | 42.75 [0.99-91.59] | 6041.71 [200.42-12086.7] | 83.24 [2.75-166.66] | 2.56 [2.26 to 2.86] |
| Southern Latin America | 632.75 [14.73-1281.23] | 135.04 [3.21-273.3] | 308.64 [6.31-631.75] | 36.36 [0.72-74] | -4.18 [-4.34 to -4.02] |
| Southern Sub-Saharan Africa | 250.12 [6.42-547.78] | 84.62 [2.36-184.55] | 477.69 [13.64-1007.39] | 76.24 [2.35-159.86] | -0.51 [-0.93 to -0.1] |
| Tropical Latin America | 874.64 [25.56-1846.97] | 83.64 [2.66-172.32] | 785.06 [17.52-1616.39] | 29.55 [0.66-60.89] | -3.6 [-3.78 to -3.43] |
| Western Europe | 2858.18 [102.79-5816.08] | 51.46 [1.84-104.89] | 1712.67 [51.36-3431.64] | 18.88 [0.56-37.51] | -3.4 [-3.5 to -3.31] |
| Western Sub-Saharan Africa | 897.97 [26.04-2093.77] | 96.23 [3.13-221.89] | 1597.55 [51.32-3410.33] | 71.67 [2.52-153.85] | -1.18 [-1.33 to -1.02] |
| Southeast Asia, East Asia, and Oceania | 16153.52 [232.38-33917.94] | 137.07 [1.96-289.09] | 27327.83 [571.33-55928.21] | 94.95 [1.93-194.28] | -1.07 [-1.2 to -0.95] |
| Central Europe, Eastern Europe, and Central Asia | 4697.18 [175.68-9653.56] | 97.33 [3.7-200.52] | 3502.82 [125.56-7088.42] | 57.55 [1.94-114.97] | -2.49 [-3.05 to -1.92] |
| High-income | 5826.06 [176.77-11890.13] | 50.62 [1.52-102.29] | 4305.27 [134.87-8960.76] | 21.42 [0.65-43.28] | -2.89 [-2.99 to -2.79] |
| Latin America and Caribbean | 1457.64 [41.12-3132.71] | 59.33 [1.86-125.15] | 1642.51 [33.45-3439.02] | 25.56 [0.52-53.64] | -3.03 [-3.13 to -2.92] |
| Sub-Saharan Africa | 2246.24 [46.42-5130.43] | 99.3 [2.4-224.86] | 4007.87 [124.27-8799.05] | 75.74 [2.7-163.97] | -1.08 [-1.16 to -0.99] |
